# Risk factors control for primary prevention of cardiovascular disease in men: Evidence from the Aragon Workers Health Study (AWHS)

**DOI:** 10.1371/journal.pone.0193541

**Published:** 2018-02-23

**Authors:** Isabel Aguilar-Palacio, Sara Malo, Cristina Feja, MªJesús Lallana, Montserrat León-Latre, José Antonio Casasnovas, MªJosé Rabanaque, Eliseo Guallar

**Affiliations:** 1 Preventive Medicine and Public Health Department, Zaragoza University, Zaragoza, Spain; 2 Aragon Institute for Health Research (IIS Aragón), Zaragoza, Spain; 3 Primary Health Care, Servicio Aragonés de Salud, Zaragoza, Spain; 4 Medicine, Psychiatry and Dermatology Department, Zaragoza University, Zaragoza, Spain; 5 Epidemiology Department, Johns Hopkins Bloomberg School of Public Health, Baltimore, United States of America; Universidad Miguel Hernandez de Elche, SPAIN

## Abstract

Benefits of cardiovascular disease (CVD) risk factors control are well known, but goals achievement remains low. The objective of this study is to evaluate the prevalence of CVD risk factors among men ina worker’s cohort with no previous CVD, to study control variations across time and the factors associated with poor control. To this end, we conducted a cohort reexamination (2010–2014) within the context of the Aragon Workers Health Study (AWHS). Data from working characteristics, analytical values and pharmacological prescription were included in the analysis. Prevalences of risk factor diagnosis and control were calculated, as well as factors associated with poor control. The prevalence of CVD risk factors was high. In 2014dyslipidaemia was the most prevalent (85.2%) followed by Hypertension (HT) (42.0%). People under treatment increased for the period analysed (p<0.001). The proportion of people treated varied from 72.2% in Diabetes Mellitus to 31.1% in dyslipidaemia in 2014. 46.2% of the workers with HT were controlled, decreasing to 21.9% in Diabetes and 11.0% in dyslipidaemia (2014). Working in a turn different to central shift was associated with poor control, especially for those working at night with HT (Odds Ratio in 2010: 3.6; Confidence Interval 95% 1.8–7.4) and dyslipidaemia (Odds Ratio 2010: 4.7; Confidence Interval 95% 1.3–16.4). We conclude that, although CVD control has increased significantly for the period studied, there are still many people that do not receive any treatment, and control goals are normally not achieved.

## Introduction

Despite the decreasing trend in the prevalence of cardiovascular disease (CVD), it remains the first cause of death in Europe, being responsible for 45% of mortality[1].

Risk factors for CVD are well established. Hypertension (HT), dyslipidaemia, Diabetes Mellitus (DM) and smoking explain up to 60% of estimated risk of CVD deaths in patients over 50 years of age[2]. The prevalence of these factors is high, even in countries with low risk of CVD: almost 50% of the Spanish population have dyslipidaemia[3]. The Darios study showed a prevalence of HT of 47% in men and 39% in women[4]. Regarding DM, the Di@bet.es study [5] estimated a prevalence of DM in Spain of 14%, with almost 6% of the patients not being aware of this diagnosis.

The benefits of CVD risk factors control are well known. The effect of pharmacological treatment to reach dyslipidaemia goals have been widely studied, and show important reductions in all-cause mortality[6]. HT control has also been associated with a decrease in incident stroke, myocardial infarction and heart failure[7], and these results improve in high risk patients as target goals become stricter[8]. Regarding DM, the intensive control of glycaemic levels has been associated with a reduction in coronary events [9]. Nonetheless, CVD risk factors are frequently uncontrolled in clinical practice[10, 11]. This low control has been observed regardless of the populations studied and the use of protocols. This low control persist even when physicians report using clinical guidelines for primary CVD prevention routinely[12]. Among the reasons to explain this low control are patient´s adherence, physicians workload or the difficult translation of guidelines into clinical practice [13].

In this context, the main objective of this study is to evaluate the prevalence of CVD risk factors among men a worker’s cohort with no previous CVD. We will analyse the achievement of control goals and the factors that are associated with poor control. Finally, we will study the prevalence of CVD risk factors and the achievement of control goals in two different years (2010 and 2014), in order to explore differences across time.

## Materials and methods

### Design

The Aragon Workers Health Study (AWHS) is a longitudinal cohort study. It started in February 2009 and enrolment was completed in December 2010. All workers were offered to participate in the study, and the response rate was 94.5%. AWHS main objective is to evaluate CVD risk factors and their association with the prevalence and progression of subclinical atherosclerosis in a Spanish middle-aged population. This study is based on the General Motors Spain automobile assembly plant (Zaragoza, Spain) and involves workers´ annual medical examinations and biological samples. Exclusion criteria include a history of CVD or the presence of clinical conditions that limit survival to less than 3 years. Female were not considered due to the low number of cases (378), as well as men <40 years old (679). Subjects who presented missing values for any of the variables included in the analysis were also excluded of the study. Further information on the AWHS can be found in the bibliography [14].

We conducted a cohort reexamination study comparing CVD risk factors control in two follow-up years: 2010 and 2014 (last follow-up year available at the present time).The final simple included 6,736 men ≥40 years old (3,400 in 2010 and 3,336 in 2014).

### Data sources and variables

Two data sources were used in this study: data obtained from the AWHS study and data from the Aragon Pharmaceutical Consumption Registry (Farmasalud).

From AWHS study we obtained information about sociodemographic and working characteristics of the individuals included in the study, lifestyles and analytical variables. These data collection at the annual medical exams is conducted by the physicians and nurses of the Medical Services of General Motors Spain, and all study procedures are described in the standard operating procedures. Data collection conforms to the ISO9001-2008 quality standard. The working characteristics considered were the number of years in the factory, work shift (rotating shift morning/afternoon, rotating shift morning/afternoon/night, central shift and night) and the type of work performed (classified as assembly line-manual, sedentary-manual, sedentary or very sedentary). This information was collected in the same way, independently of the working characteristics. Information about smoking status (former smoker, current smoker and non-smoker) was self-reported, and body mass index and waist circumference were objectively collected and included in the analyses. Each participant also provided a sample of blood and urine after overnight (>8 h) fasting for laboratory analyses. Fasting serum glucose and cholesterol are measured by spectrophotometry (Chemical Analyzer ILAB 650, Instrumentation Laboratory). Whole blood HbA1c is measured by reverse-phase cationic exchange chromatography and quantification by double wave-length colorimetry quantification (Analyzer ADAMS A1c HA-810, Arkray Factory). This item was only available for 2010. Systolic Blood Pressure and Diastolic Blood Pressure is measured three consecutive times using an automatic oscillometric sphygmomanometer OMRON M10-IT (OMRON Healthcare Co. Ltd., Japan) with the participant sitting after a 5-min rest. Self-reported treatments were also evaluated. Finally, a subgroup of the cohort (40 to 55 years old) is selected for an intensive follow-up. In this group, the presence of carotid plaque in both carotid arteries is determined using ultrasound images and nutritional questionnaires are conducted. Belonging of the worker to this intensive follow-up group was also considered.

On the other hand, information on drug preventive treatments was obtained from Farmasalud. This database collects information on drugs dispensed by pharmacies in prescriptions issued via the Aragon Health System, but does not record drugs obtained without a prescription, or prescriptions from private healthcare providers or hospitals. Prescription data is collected using ATC codes (Anatomical Therapeutic Chemical Classification System, as defined by the World Health Organization)[15]. The ATC codes included were: A10 (antidiabetics), C02, C03, C07, C08, C09 (drugs with antihypertensive effect), and C10 (lipid-lowering drugs). These data on drug prescribing were combined with anonymized data collected from the AWHS cohort. The criteria to consider a patient as treated was the existence of at least one drug prescription for HT, dyslipidaemia or DM during the year analysed (2010 or 2014).

### Analyses

Descriptive analyses were conducted to know sociodemographic, working, lifestyles, clinical and treatment characteristics. Results were stratified by year of analysis and statistical tests were applied to explore time differences.

Secondly, CV risk factors prevalence (HT, DM and dyslipidaemia) was obtained for the studied cohort. Workers were considered as having a risk factor if they fulfil the 6^th^ European Guideline criteria [16] or if they had a preventive drug treatment (self-reported or registered in Farmasalud database). Thus, HT criteria were Systolic Blood Pressure ≥140 mmHg or Diastolic Blood Pressure ≥90 mmHg or existence of HT preventive drug treatment. Workers were classified as diabetic if HbA1c was ≥6.5% or Fasting Plasma Glucose≥126 mg/dL, or when at least one drug treatment for DM was observed. Finally, dyslipidemia was diagnosed when total cholesterol ≥190 mg/dL or low-density lipoprotein cholesterol ≥115 mg/dL or drug treatment for dyslipidemia existed (when workers had been diagnosed of DM, this values decreased to total cholesterol ≥175 mg/dL or low-density lipoprotein cholesterol ≥100 mg/dL) low-density lipoprotein cholesterol was calculated using Friedewald´s estimation[17]. For each major risk factor, assessment of treatment goals achievement was considered, according to 6^th^ European Guideline. Chi square tests were conducted to calculate prevalence differences between 2010 and 2014.

Finally, logistic regression analyses were developed to explore variables associated with risk factors control. Two models were conducted for each year analysed and for each risk factor. Model 1 shows results adjusted by age, working conditions, lifestyles, diagnosis and follow-up. Model 2 added the effect of preventive treatment, taking into consideration those who received at least one prescription in that year. Adjustment variables were selected among those variables available that, according literature, have an effect on risk factors control. Odds Ratios (OR) and their 95% Confidence Intervals (95%CI) were calculated. Statistical analyses were conducted using Stata 12®.

### Ethical aspects

All factory workers signed consent to participate in AWHS. In this consent they allow the use of annual health exam data and the provision of blood samples, as well as the use of other medical records in order to tackle the objectives of the study. This study was approved by the Aragon Research Ethics Committee.

## Results and discussion

### Results

The main characteristics of the AWHS cohort for both years analysed are available in Table 1. The median of age was 55.2 years in 2014. The majority of the workers had a rotating shift morning/afternoon (58.1%) and works in the assembly line (87.2%). Regarding lifestyles, current smokers decreased from 35.2% in 2010 to 30.1% in 2014. The values of Body Mass Index, waist circumference and obesity did not vary for the years analysed. We observed a statistically significant decreased in cholesterol values, both total and low-density lipoprotein cholesterol, Fasting Plasma Glucose and blood pressure (p<0.001). Variations in HbA1c could not be evaluated because this information was not available in 2014. On the contrary, the prevalence of CVD preventive treatment increased, especially in the case of dyslipidaemia drug treatment (from 15.4% in 2010 to 27.2% in 2014). Finally, the percentage of workers with an intensive follow-up in the cohort increased from 59.4% in 2010 to 66.6% in 2014 (p<0.001).

**Table 1:** AWHS cohort descriptive.

Variables	2010 (N = 3400)	2014 (N = 3336)	p
Years of age, median;Q1-Q3	51.9; 49.0–55.0	55.2; 51.7–57.8	<0.001
Years in the factory, median;Q1-Q3	27.6; 22.6–28.1	31.5; 26.4–31.9	<0.001
Work Shift, %			0.948
Rotating shift morning/afternoon	58.1	58.1	
Rotating shift morning/afternoon/night	21.7	21.2	
Central shift	8.8	8.8	
Night	11.5	11.8	
Work Type, %			0.623
Assembly line-manual	87.2	87.9	
Sedentary	12.6	12.0	
Very sedentary	0.2	0.1	
Smoking, %			<0.001
Former smoker	42.2	47.0	
Current smoker	35.2	30.1	
Non smoker	22.7	22.9	
Body mass index (BMI), median;Q1-Q3	27.6; 25.6–29.9	28.0; 25.5–30.0	0.092
Waist circumference (WC), median;Q1-Q3	97.5; 91.7–103.9	97.4; 91.3–104.0	0.999
Obesity, %	35.0	35.6	0.636
Total cholesterol (mmol/L), median;Q1-Q3	5.6; 5.0–6.2	5.3; 4.8–5.9	<0.001
LDL cholesterol (mmol/L), median;Q1-Q3	3.5; 2.9–4.0	3.3; 2.8–3.8	<0.001
Fasting Plasma Glucose (mmol/L), median;Q1-Q3	5.4; 5.0–5.9	5.2; 4.8–5.7	<0.001
HbA1c (%)	5.4; 5.3–5.6	NA	NA
Systolic Blood Pressure (mmHg), median;Q1-Q3	126.0; 117.0–136.0	124.0; 115.0–134.0	<0.001
Diastolic Blood Pressure (mmHg), median;Q1-Q3	85.0; 78.0–91.0	81.0; 75.0–87.0	<0.001
Cardiovascular preventive treatment, %			
Hypertension drug treatment	24.4	29.6	<0.001
Type 2 Diabetes drug treatment	4.6	6.1	0.004
Dyslipidaemia drug treatment	15.4	27.2	<0.001
Intensive follow-up, %	59.4	66.6	<0.001

Obesity: BMI > = 30 or WC> = 102 cm in men; NA no application (missing variable); p: statistical significance; Q1-Q3: quartile1-quartile3; Treatment: declared or registered in Pharmacy database.

In Table 2, information about risk factor diagnosis, treatment and control is available. The prevalence of CVD risk factors remained stable for the period analysed. Dyslipidaemia was the most prevalent risk factor (85.2% in 2014). For all the risk factors considered, the people under treatment increased (p<0.001), as well as the knowledge of people´s own treatment (p<0.001). DM showed the highest prevalence of workers treated (72.2% in 2014). On the other hand, only 31.1% of the workers with dyslipidaemia were under treatment in 2014. Regarding risk factors control, 46.2% of the workers with HT were controlled for this risk factor. This percentage decreased to 21.9% in DM (Fasting Plasma Glucose levels) and 11.0% in dyslipidaemia (total and low-density lipoprotein cholesterol levels). When only people treated were considered, these percentages increased to 65.5% in the case of HT, 28.9% in DM and 34.5% in dyslipidaemia.

**Table 2:** Diagnosis and risk factor control.

		2010 (N = 3400)	2014 (N = 3336)	p
**Hypertension**	HT diagnosis N (%)	1502 (44.2)	1400 (42.0)	0.067
	Pharmacological treatment according EMR N (%)	817 (54.4)	967 (69.1)	<0.001
	Pharmacological treatment self-declared N (%)	624 (41.5)	893 (64.6)	<0.001
	Controlled HT (SBP<140 and DBP<90) N (%)	397 (26.4)	647 (46.2)	<0.001
	Controlled HT among treated (SBP<140 and DBP<90) N (%)	397 (47.8)	647 (65.5)	<0.001
**Diabetes Mellitus**	DM diagnosis N (%)	271 (8.0)	270 (8.1)	0.853
	Pharmacological treatment according EMR N (%)	147 (54.2)	195 (72.2)	<0.001
	Pharmacological treatment self-declared N (%)	123 (45.4)	170 (63.2)	<0.001
	Controlled HbA1c (<7) N (%)	198 (73.3)	NA	NA
	Controlled FPG (<110 mg/dl) N (%)	27 (10.0)	59 (21.9)	<0.001
	Controlled HbA1c and FPG (HbA1c<7 and FPG <110) N (%)	20 (7.4)	NA	NA
	Controlled HbA1c among treated (HbA1c<7) N (%)	93 (60.4)	NA	NA
	Controlled FPG among treated (<110 mg/dl) N (%)	22 (14.2)	59 (28.9)	0.001
	Controlled among treated (HbA1c<7 and FPG <110) N (%)	15 (9.7)	NA	NA
**Dyslipidaemia**	Dyslipidaemia diagnosis N (%)	2857 (84.0)	2841 (85.2)	0.198
	Pharmacological treatment according EMR N (%)	509 (17.8)	882 (31.1)	<0.001
	Pharmacological treatment self-declared N (%)	317 (11.3)	714 (25.5)	<0.001
	Controlled TC (<190 mg/dl)* N (%)	208 (7.3)	454 (16.0)	<0.001
	Controlled LDL (<115 mg/dl)* N (%)	287 (10.0)	592 (20.8)	<0.001
	Controlled TC and LDL (C<190 and LDL<115)* N (%)	95 (3.3)	313 (11.0)	<0.001
	Controlled TC among treated (<190 mg/dl)* N (%)	103 (19.6)	334 (36.8)	<0.001
	Controlled LDL among treated (<115 mg/dl)* N (%)	139 (26.5)	440 (48.5)	<0.001
** **	Controlled TC and LDL among treated (C<190 and LDL<115)* N (%)	95 (18.1)	313 (34.5)	<0.001

DBP: Diastolic Blood Pressure; DM: Diabetes Mellitus; EMR: Electronic Medical Records; FPG: Fasting Plasma Glucose; HT: Hypertension; LDL: Low Density Cholesterol; N: number; NA: no application; SBP: Systolic Blood Pressure; TC: Total Cholesterol; Diagnosis criteria: HT: SBP> = 140 or DBP> = 90 or drug treatment for HT; DM: HbA1c> = 6.5 or FPG> = 126 or drug treatment for DM

Dyslipidaemia: TC> = 190 or LDL> = 115 or drug treatment for dyslipidaemia (TC> = 175 or LDL> = 100 if DM).

* TC<175 and LDL<100 in patients with DM.

Patients treated: patients who self-declared treatment or received at least one prescription in that year.

**Fig 1:**
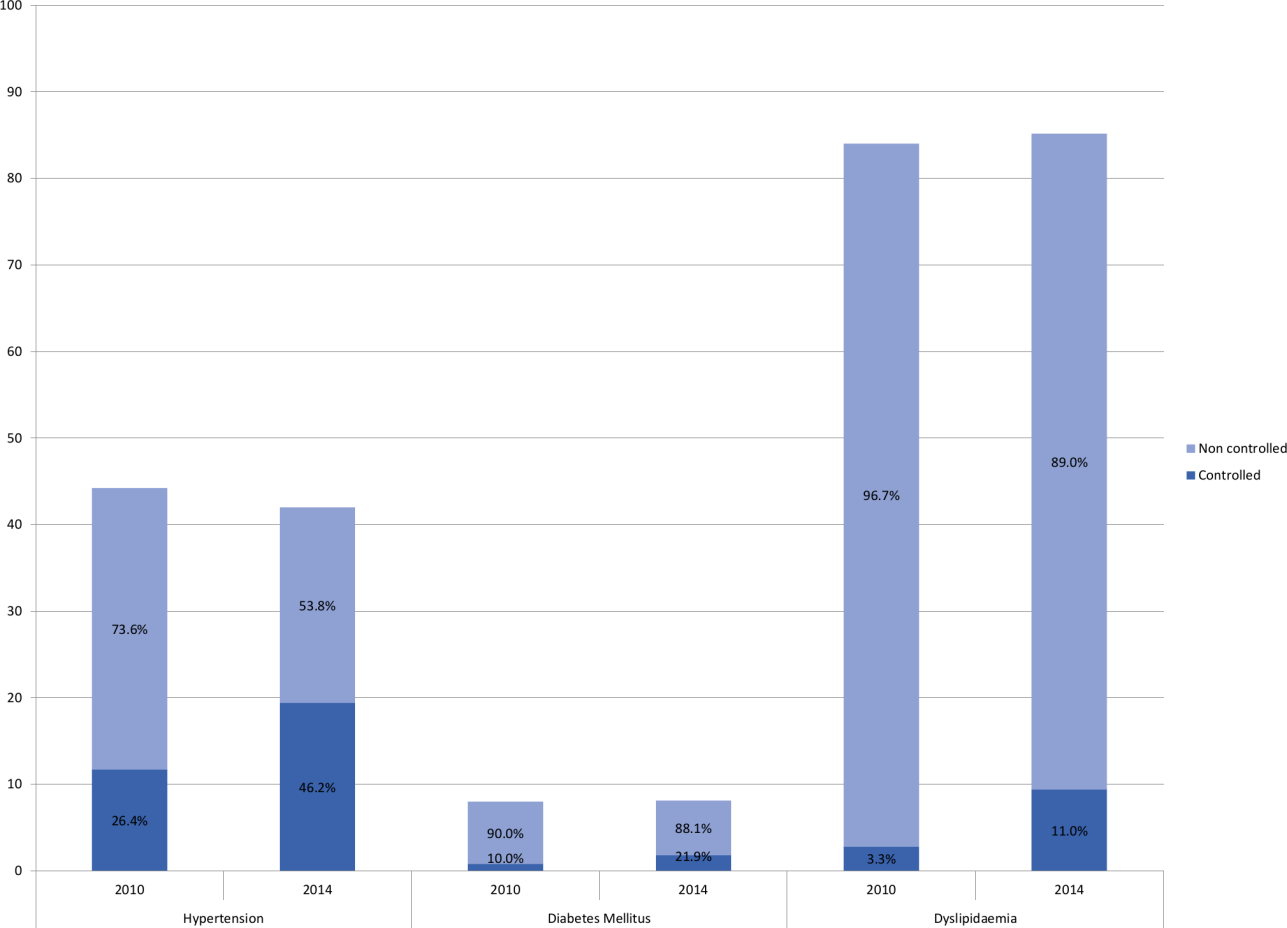
Cardiovascular risk factors prevalence and control for the period studied. Each bar shows the total prevalence of disease in the year indicated. Percentages in each bar correspond to the proportion of subjects controlled and non-controlled of all those diagnosed.

Fig 1 shows the proportion between controlled and uncontrolled patients, showing an important control increase for all the risk factors during the period analysed.

Tables 3 and 4 show the characteristics associated with low risk factors control. Work shift, work type, Body Mass Index and HT drug treatment were associated with HT control. Regarding work shift, people working in a turn different to the central shift showed higher risk of bad HT control. This relationship was statistically significant for both years considered and was especially strong when working at night (OR in 2010: 3.64; CI95% 1.79–7.40). Sedentary work was also a risk factor of bad HT control in 2014 (OR: 1.91; CI95% 1.19–3.06) but this association was not shown in 2010. Also, the increase of Body Mass Index was associated with a higher risk of bad HT control in 2010. Finally, for both years analysed, HT drug treatment decreased the risk of bad HT control (OR in 2010: 0.01; CI95% 0.01–0.02).

**Table 3:** Characteristics associated with poor control of hypertension (HT) and diabetes mellitus (DM), among people with a diagnosis or HT or DM.

	Control HT OR (CI95%)				Control DM OR (CI95%)			
	2010 (N = 1502)		2014 (N = 1400)		2010 (N = 271)		2014 (N = 270)	
	Model 1	Model 2	Model 1	Model 2	Model 1	Model 2	Model 1	Model 2
Age	0.95 (0.92–0.99)*	1.00 (0.95–1.05)	0.95 (0.92–0.99)*	1.03 (0.98–1.08)	1.01 (0.88–1.16)	1.00 (0.87–1.15)	0.99 (0.89–1.11)	1.01 (0.90–1.13)
Years in the factory	1.02 (0.99–1.06)	1.02 (0.98–1.07)	1.01 (0.97–1.04)	1.00 (0.96–1.04)	1.10 (0.99–1.22)	1.11 (0.99–1.23)	1.03 (0.93–1.14)	1.04 (0.93–1.16)
Work Shift								
Rotating morning/afternoon	2.95 (1.79–4.85)*	2.79 (1.51–5.14)*	1.93 (1.17–3.19)*	1.90 (1.05–3.43)*	0.43 (0.03–6.49)	0.47 (0.03–7.53)	1.11 (0.21–5.83)	1.21 (0.23–6.42)
Rotating morning/afternoon/night	2.50 (1.51–4.16)*	2.31 (1.25–4.29)*	1.68 (1.00–2.81)*	1.63 (0.88–3.01)	0.33 (0.02–5.23)	0.34 (0.92–5.66)	0.97 (0.17–5.38)	0.94 (0.17–5.35)
Central shift	1	1	1	1	1	1	1	1
Night	3.77 (2.09–6.79)*	3.64 (1.79–7.40)*	2.94 (1.66–5.20)*	2.63 (1.33–5.21)*	0.06 (0.00–0.98)*	0.06 (0.00–1.10)	0.21 (0.04–1.15)	0.25 (0.04–1.42)
Work Type								
Assembly line	1	1	1	1	1	1	1	1
Sedentary	1.48 (0.96–2.27)	1.50 (0.90–2.48)	1.78 (1.18–2.70)*	1.91 (1.19–3.06)*	1.00 (0.14–7.17)	1.02 (0.13–7.84)	0.78 (0.24–2.56)	0.66 (0.19–2.27)
Current smoker	1.20 (0.93–1.54)	1.11 (0.82–1.50)	1.00 (0.78–1.27)	1.08 (0.81–1.46)	1.36 (0.54–3.42)	1.39 (0.55–3.52)	0.98 (0.49–1.97)	0.86 (0.42–1.76)
Body mass index (BMI)	1.08 (1.01–1.15)*	1.10 (1.02–1.19)*	1.05 (1.00–1.12)	1.05 (0.97–1.12)	0.95 (0.75–1.21)	0.95 (0.75–1.21)	1.06 (0.89–1.26)	1.05 (0.88–1.26)
Waist circumference (WC)	0.97 (0.94–0.99)*	0.99 (0.96–1.01)	0.98 (0.96–1.00)*	0.99 (0.97–1.02)	1.04 (0.95–1.14)	1.04 (0.95–1.14)	1.00 (0.93–1.07)	1.00 (0.93–1.07)
Hypertension	NA	NA	NA	NA	0.82 (0.30–2.23)	0.56 (0.17–1.90)	0.53 (0.24–1.15)	0.70 (0.23–2.11)
Diabetes Mellitus	0.87 (0.61–1.24)	1.67 (0.89–3.14)	1.02 (0.74–1.41)	2.01 (0.95–4.24)	NA	NA	NA	NA
Dyslipidaemia	0.91 (0.64–1.31)	0.79 (0.52–1.21)	1.09 (0.78–1.54)	1.17 (0.75–1.83)	1.62 (0.39–6.66)	1.89 (0.42–8.49)	2.59 (0.86–7.73)	2.95 (0.85–10.26)
Intensive follow-up	0.97 (0.71–1.32)	1.10 (0.77–1.58)	0.65 (0.50–0.86)*	0.74 (0.53–1.03)	0.85 (0.29–2.48)	0.82 (0.29–1.98)	4.78 (0.22–1.04)	0.52 (0.23–1.15)
Hypertension drug treatment	-	0.01 (0.01–0.02)*	-	0.02 (0.01–0.03)*	-	2.05 (0.67–6.21)	-	0.83 (0.32–2.18)
Type 2 Diabetes drug treatment	-	0.59 (0.28–1.27)	-	0.63 (0.28–1.44)	-	0.76 (0.29–1.98)	-	0.30 (0.11–0.78)*
Dyslipidaemia drug treatment	-	0.79 (0.52–1.21)	-	0.90 (0.67–1.20)	-	0.67 (0.25–1.76)	-	0.66 (0.31–1.41)

Model 1: adjusted by age, years in the factory, work shift, work type, smoker, BMI, wc, HT, DM, dyslipidemia and follow-up; Model 2: variables included in model 1 + treatment.

*results statistically significant; NA: no application.

Control DM: taking into consideration FPG; Patients treated: patients who received at least one prescription in that year.

**Table 4:** Characteristics associated with poor dyslipidaemia control, among people with a diagnosis of dyslipidaemia.

	Control dyslipidaemia OR (CI95%)			
	2010 (N = 2857)		2014 (N = 2841)	
	Model 1	Model2	Model 1	Model2
Age	0.93 (0.87–1.00)*	0.97 (0.90–1.05)	0.90 (0.87–0.94)*	0.97 (0.93–1.02)
Years in the factory	1.05 (1.00–1.11)	1.07 (1.01–1.14)*	1.02 (0.98–1.05)	1.02 (0.98–1.06)
Work Shift				
Rotating morning/afternoon	1.75 (0.71–4.28)	1.86 (0.71–4.89)	1.32 (0.79–2.21)	1.38 (0.77–2.46)
Rotating morning/afternoon/night	2.95 (1.10–7.91)*	2.78 (0.96–8.06)	1.78 (1.02–3.09)*	1.61 (0.86–3.01)
Central shift	1	1	1	1
Night	3.83 (1.17–12.58)*	4.68 (1.33–16.42)*	1.54 (0.84–2.82)	1.61 (0.81–3.21)
Work Type				
Assembly line	1	1	1	1
Sedentary	2.12 (0.85–5.30)	1.92 (0.74–4.97)	1.23 (0.77–1.97)	1.06 (0.63–1.78)
Current smoker	1.28 (0.81–2.04)	1.14 (0.68–1.90)	0.89 (0.68–1.16)	0.92 (0.67–1.25)
Body mass index (BMI)	0.92 (0.82–1.04)	0.92 (0.80–1.06)	1.02 (0.95–1.09)	1.06 (0.97–1.15)
Waist circumference (WC)	1.02 (0.97–1.06)	1.04 (0.98–1.10)	0.99 (0.96–1.01)	0.98 (0.95–1.01)
Hypertension	0.46 (0.29–0.75)*	0.63 (0.32–1.23)	0.49 (0.38–0.64)*	1.08 (0.64–1.81)
Diabetes Mellitus	0.32 (0.19–0.52)*	3.14 (0.71–13.79)	0.53 (0.38–0.75)*	3.10 (1.04–9.23)*
Dyslipidaemia	NA	NA	NA	NA
Intensive follow-up	0.87 (0.50–1.50)	1.13 (0.62–2.04)		1.11 (0.77–1.59)
Hypertension drug treatment	-	0.85 (0.45–1.62)	-	0.51 (0.31–0.84)*
Type 2 Diabetes drug treatment	-	0.14 (0.03–0.65)*	-	0.29 (0.09–0.91)*
Dyslipidaemia drug treatment	-	0.01 (0.00–0.02)*	-	0.01 (0.00–0.01)*

Model 1: adjusted by age, years in the factory, work shift, work type, smoker, BMI, wc, HT, DM, dyslipidemia and follow-up; Model 2: variables included in model 1 + treatment.

*results statistically significant; NA: no application.

Patients treated: patients who received at least one prescription in that year.

When DM control was studied, the existence of DM drug treatment improved its control, but this effect was only observed in 2014 (OR: 0.30; CI95% 0.11–0.78). People working at night also showed better DM control than those working in a central shift in 2010, but this association was not statistically significant after adjustment by drug treatments.

Regarding dyslipidaemia (Table 4), its control increased as the workers grow older (OR: 0.90 in 2014; CI95% 0.87–0.94) and when HT and DM diagnosis coexist. On the other hand, rotating morning/afternoon/night was associated with a poor control for both years analysed, respect to central shift. After drug treatments adjustment, the number of years in the factory (OR: 1.07; CI95% 1.01–1.14) and working at night in relation to having a central shift (OR: 4.68; CI95% 1.33–16.42) were associated statistically with a poor control of dyslipidaemia. In 2010, the existence of drug treatment for DM and dyslipidaemia increased the control of this risk factor. In 2014, a better control existed when patients have drug treatment for any CVD risk factor. On the contrary, a diagnosis of DM was associated with higher risk of low dyslipidaemia control (OR: 3.10; CI95% 1.04–9.23). The belonging to the intensive follow-up group was not associated with any risk factor control.

### Discussion

The prevalence of CVD risk factors in our cohort remains stable for the five years analysed and shows a high prevalence: almost half of the workers have HT, 85% have been classified as having dyslipidaemia and 8% have DM. These results show some variations when comparing with other studies. The EURIKA study[11] showed a higher proportion of people with HT and DM than our study (72.7% and 26.8% respectively) but only 57.7% of the population was diagnosed with dyslipidaemia. In Spain, the ERICE study[3] found similar prevalences of HT and DM (37.6% and 6.2% respectively) although dyslipidaemia prevalence was lower (46.7%). Differences could be explained because ERICE dyslipidaemia diagnosis criteria is total cholesterol >200 mg/dL, instead of 190, and they also include younger participants.

Pharmacological treatment and risk factors control have increased significantly during the period analysed for all the CVD risk factors studied. It is remarkable in the case of dyslipidaemia, where treatment prevalence almost double in 5 years (from 17.8% in 2010 to 31.1% in 2014) and people controlled among those treated for dyslipidaemia increased from 18.1% to 34.5%. The effect of study enrolment on participant´s behaviour has been attributed to the fact of being observed or the increasing awareness of the participant about a health problem (Hawthorne effect) [18, 19]. In the case of CVD, this effect has already been described. In ARTPER cohort [20] an improvement in risk factors control in 5 years of follow-up was found, with the only exception of DM. Paradoxically, in the AWHS cohort, belonging to the intensive follow-up group was not associated with a better control. This lack of effect could be explained because risk factors control and pharmacological prescription are usually carried out by the occupational health service and Primary Care doctors, and this attention is the same for all the workers included in the study. The role of being subjected to ultra-specialized techniques seems to have no influence on people´s risk factors goals achievement.

According to clinical guidelines, there are still many workers that should have pharmacological treatment but remain untreated. This is especially important in the case of dyslipidaemia. We calculated the SCORE (European SCORE for low CVD countries [21]) of untreated people with dyslipidaemia, in order to evaluate their CVD risk. The mean of 10-year risk of fatal CVD in this group was 1.4% and only 2.5% of these workers were considered as presenting high risk levels (≥5% of risk of fatal CVD within a 10 year period). Due to its low proportion of workers in high risk, other control strategies, like diet or physical activity recommendations, could be being used in these patients.

Although the proportion of people treated has increased, the achievement of control goals is still low. Only 65% of the workers treated for HT are controlled and this percentage decrease to 34.5% in dyslipidaemia and 28.9% in DM. These results are consistent with literature. The Darios study found that, when European guideline criteria are applied, there is almost no dyslipidaemia control[22]. The EURIKA study showed also a poor risk factors control in primary prevention (6): less than half of HT and dyslipidaemic patients achieved treatment goals, and only one-third of patients with DM. Banegas et al.[23] got poorer results in a Spanish population, with only 22.7% of all patients with HT being under controlled. This percentage increased up to 48.5% when only people aware of their diagnosis were considered. On the contrary, other study conducted in Spain [24], showed a 71% of aware diabetic patients that achieved HbA1c control goal. Despite of the high level of DM treatment control, general achievement of cardio metabolic goals, especially lifestyles, was poor. Therefore, it seems clear that the existence of pharmacological treatment is not necessary associated to a proper control and other factors, like patient´s adherence, should be considered. It has been estimated that up to 9% of European CVD events could be attributed to low adherence to pharmacological treatments[25]. A study conducted in Italy evaluating pharmacological treatment for HT [26] reported a significant decreased of CVD events in patients with high levels of adherence respect to those with poor adherence to treatment. Adherence to statins has also demonstrated a reduction of nonfatal coronary artery disease[27]. In spite of its importance, adherence to CVD treatment is usually low, especially in primary prevention[28]. Finally, other aspects of CVD risk factors approach should be properly addressed, as lifestyles interventions, which in some cases have shown even better results than pharmacological therapies[29].

The main factor associated with poor CVD risk factor control was working on a shift different than central one, especially working at night. There is evidence in literature in favour of an association between shift work and CVD [30, 31] and has been attributed to circadian rhythms, stress, behaviour and biochemical changes, among others. Nonetheless, the influence of shift on risk factor achievement goals has not been previously evaluated, but it is probably related with the same factors than the appearance of CVD. To mitigate the health effects of night shift working, several interventions, like shift schedule or the promotion of physical activity and healthy diet, has shown positive effects[32]. On the contrary, the presence of pharmacological treatment increased drastically the achievement of treatment goals, as it was expected. In the case of dyslipidaemia, it is interesting that being treated for DM improved dyslipidaemia control, but the diagnosis of DM was associated with a poorer dyslipidaemia control. Due to the low precision of this result (CI95% 1.04–9.23) this result should be interpreted cautiously.

This study has some limitations. The main restriction is the population included in the cohort. The low number of women and young people did not allow us to analyse these groups, which limits the generalization of our results. Other limitation is the lack of analytical values for HbA1c in 2014. Because of this, treatment goals for DM in 2014 could only be calculated using Fasting Plasma Glucose. The possibility of Hawthorne effect, in which participants change their behaviour when being observed, should also be considered. Nonetheless, the effect of belonging to the intensive follow-up group on risk factors control was not significant. Finally, we classified patients as having pharmacological treatment when they had received at least one prescription of this pharmacological group. Although CVD treatments are long term, this classification criterion could have produced some bias, and made not possible to evaluate adherence to treatment. On the other hand, the existence of a workers cohort allowed us to follow participants throughout time. The use of different data sources, both clinical and administrative, provided a wide perspective of CVD primary prevention. Finally, as far as we know, this is the first study that evaluates the influence of working factors on CVD control.

## Conclusions

The enrolment of participants in a cohort could have improved AWHS CVD management for the period analysed. On the contrary, shift work was associated with a poor control. Although CVD risk factors control has increased for the period analysed there is still space for improvement. There are many patients that do not receive any treatment even when they need it, and control goals are not frequently achieved. A closer patient´s control, the evaluation of pharmacological adherence or the adjustment of goals to our target population should be aspects to work on. Finally, when longer follow-up is completed, the effect of goal achievement on the incidence of CVD events should be evaluated. This is fundamental in order not to lose sight of the final objective of all these measures: preventing CVD appearance.
