# Correction: Risk factors control for primary prevention of cardiovascular disease in men: Evidence from the Aragon Workers Health Study (AWHS)

**DOI:** 10.1371/journal.pone.0228906

**Published:** 2020-02-04

**Authors:** Isabel Aguilar-Palacio, Sara Malo, Cristina Feja, MªJesús Lallana, Montserrat León-Latre, José Antonio Casasnovas, MªJosé Rabanaque, Eliseo Guallar

In the Funding section, the information provided is incomplete. The complete, correct Funding section is:

This work was supported by the Instituto de Salud Carlos III and Fondo Europeo de Desarrollo Regional (FEDER). PI13/01668 (http://www.isciii.es).
